# Seroprevalence and genotyping of avian infectious bronchitis virus detected from Iranian unvaccinated backyard chickens

**Published:** 2018-02

**Authors:** Shima Shokri, Vahid Karimi, Arash Ghalyanchi Langeroudi, Mehdi Vasfi Marandi, Masoud Hashamzadeh, Taha Zabihipetroudi, Hamideh Najafi, Farshad Tehrani

**Affiliations:** 1 Department of Clinical Sciences, Faculty of Veterinary Medicine, University of Tehran, Tehran, Iran; 2 Department of Microbiology and Immunology, Faculty of Veterinary Medicine, University of Tehran, Tehran, Iran; 3 Razi Vaccine and Serum Research Institute, Agricultural Research and Extension Organization, Tehran, Iran; 4 Department of Pathobiology, School of Veterinary Medicine, Shiraz University, Shiraz, Iran; 5 Department of Health and Management of Poultry Diseases, Iranian Veterinary Organization, Tehran, Iran

**Keywords:** Avian infectious bronchitis, Backyard chicken, Phylogenetic, Spike, Iran

## Abstract

**Background and Objectives:**

Different epidemiological studies have found that backyard chickens are a reservoir for poultry diseases. Most backyard chicken flocks have a poor level of biosecurity, which increases the risk of spread of diseases. In recent years, the number of backyard chickens has been on the rise in Iran. However, the health status of backyard flocks is still poorly documented. Thus, this study aimed at examining the seroprevalence of antibodies against infectious bronchitis virus (IBV) and molecular surveillance and genotyping of IBV among backyard chickens (without vaccination history) in Mazandaran province, North of Iran, 2014.

**Materials and Methods:**

A total of 460 blood samples of unvaccinated backyard chickens in the mentioned area were tested for antibodies against IBV using commercial ELISA. Also, cecal tonsils were collected from 75 chickens in the same area. Real time RT-PCR (for detection) and RT-PCR and sequencing spike gene were performed.

**Results:**

The seropositivity rate was 54.5%. In addition, we detected 793/B, Variant 2, and QX in the backyard flocks and performed phylogenetic studies on them. The phylogenetic study revealed that the detected genotypes had high homology with IBV strains that were infected broilers, pullets, and layers in Iran.

**Conclusion:**

There is a need for continuous monitoring of IBV among avian species to complete the epidemiological map and work on the pathogenesis of Iranian IBV strains in Iranian backyard chickens.

## INTRODUCTION

Avian infectious bronchitis virus (IBV) belongs to the genus *Gammacoronavirus* of the *Coronaviridae* family and is the etiologic agent of infectious bronchitis (IB), which is a major, highly complex infectious disease of poultry caused by multiple serotypes of IBV (1). IBV possesses a single-stranded positive-sense RNA genome (approximately 27.6 kb) encoding 4 structure proteins (phosphorylated nucleocapsid (N) protein, small envelope protein (E), integral membrane glycoprotein (M), and spike glycoprotein (S)) in the order of 5′-Pol-S-3a-3b-E-M-5a-5b-N-UTR-3′ (2). The S glycoprotein is cleaved into S1 and S2 subunits posttranslationally. S1 protein involves in infectivity, contains serotype-specific sequences, hemagglutinin activity, and virus neutralizing epitopes. The mutations, deletions, insertions, and recombination events that have been observed in multiple structural genes, especially in the S1 gene, of IBV isolates recovered from natural infections have been considered to contribute to the genetic diversity and evolution of IBV, and consequently, to the development of a number of IBV serotypes (3, 4). IB affects chickens of all ages, and IBV replicates primarily in the respiratory tract and in some epithelial cells of the kidney, gut and oviduct, resulting in reduced performance, reduced egg quality and quantity, increased susceptibility to infections with other pathogens, and condemnations at processing. IBV is a major poultry pathogen that is endemic worldwide and leads to serious economic losses (5, 6). IB has been reported in peafowl, teal, partridge, turkey, pheasant, racing pigeon and guinea fowl (7). Therefore, serological and molecular characterization of the field isolates of the IBV is highly important. IB was firstly described in North Dakota, USA, in 1930 (8). The first isolation of IBV in Iran was reported by Aghakhan et al. in 1994. The isolate showed the antigenic relationship to the mass serotype (9). IB is still a serious problem in Iran. Some newly emerging IBV isolates have recently been found. Backyard chicken is considered an important source of spread and persistence of different diseases (IB, Newcastle disease and avian influenza) among the chickens in poultry farms, playing a major role in the epidemiology of avian infectious diseases. Most household flocks are small and of mixed age and feed mainly by scavenging. Chickens from different households may mix, potentially exposing them to different diseases.

Moreover, no preventive and controlling strategy has been undertaken against IB in backyard chickens in Iran (10–12).

## MATERIALS AND METHODS

### Study area and sampling.

Mazandaran province is one of the 31 provinces of Iran and is located along the Caspian Sea in Iran’s Region 3, just east of Gilan province, west of Golestan province, and north of Tehran and Semnan provinces (36.5656°N 53.0588°E). Mazandaran is a major producer of poultry, and poultry farmers in this region provide an important economic addition to the traditional dominance of agriculture. For serology, we collected 460 sera from backyard chickens (9 cities, Table 1) during October to December 2014; and for molecular detection and characterization, we collected cecal tonsils from 75 chickens.

**Table 1.** Seroprevalence of avian infectious bronchitis viruses (IBV) antibody (ELISA assay, Biocheck) in unvaccinated backyard chickens in Mazandaran province, Iran, 2014.

**City**	**No. of. Samples**	**Positive (Percent)**	**Mean Titer**
Behshar	32	93.75	7115
Sari	89	98.80	7597
Jouybar	48	100	7211
Neka	60	31.7	6171
Nour	24	58.4	1866
Babolsar	28	46.4	3745
Ghaemshahr	52	21.2	2835
Amol	67	14.9	1373
Babol	60	30	6629
Total & Average	460	54.5%	4949

### ELISA.

Poultry sera were assayed for IBV antibodies using a commercially available blocking ELISA (Biocheck), and antibody titers obtained from samples were also evaluated. Laboratory results of IBV ELISA were entered and managed using Microsoft Excel (Windows 2010). Descriptive statistics for the ELISA antibody titers were performed using the same program.

### RNA extraction.

Viral genomes were extracted from cecal tonsil samples (CinnaPure RNA, Sinaclone, Iran) according to manufacturer’s instructions. The extracted viral RNA was contained in a 1.5 mL sterile RNase and DNase- free microtube and stored at −70°C until further use.

### cDNA synthesis.

Viral RNA was reverse transcribed using Revert Aid First Strand cDNA Synthesis Kit (Thermo Scientific); cDNAs were stored at −20°C until use.

### Real time PCR for IBV detection.

Real time RTPCR was conducted in a QIAGEN Rotor-Gene Q (Corbett Rotor-Gene 6000) (USA, CA). Forward primer (IBV5′GU391 5′-GCTTTTGAGCCTAGCGTT-3′), reverse primer (IBV5′GL533 5′GCCATGTTGTCACTGTCTATTG-3′), and Taqman® dual-labeled probe (IBV5′G probe 5′-FAMCACCACCAGAACCTGTCACCTC-BHQ1-3′) were used to amplify and detect a 143-bp fragment at the 5′UTR of the IBV genome (13).

### Partial S1 amplification for genotyping.

A pair of degenerated primers (SX1: CACCTAGAGGTTTGYTWGCATG and SX2: TCCACCTCTATAAACACCYTTAC) and 3 others (SX3: TAATACTGGYAATTTTTCAGATGG& SX4: AATACAGATTGCTTACAACCACC) were finally selected for use in the initial PCR and in the subsequent nested PCR (14). First round amplification was performed in a final volume of 20 μL (2 μL D.W, 13 μL Sinaclon 2X PCR master mix (Sinaclon, Iran), 2 μL of SX1 and SX2 primers and 3 μL of cDNA; amplification was performed with a thermal profile (94° C for 2 min, 94° C for 15 sec, 58° C for 30 sec, 72° C for 30 sec, and 72° C for 10 min) for 35 cycles. Amplifications were performed in an Eppendorf master cycler gradient thermocycler (Eppendorf, Hamburg, Germany). Nested-PCR reactions (20 uL) were performed using 1 uL of the first PCR product. The reaction mixture was the same as the previous PCR using nested primers (SX3 & SX4). The reaction products were analyzed by electrophoresis in 1.5% agarose gels in TAE buffer, stained with GelRed™ (Biotium, USA) and visualized under UV light.

### Sequencing and bioinformatics analysis.

The PCR products of all positive samples were purified using PCR Purification Kit (Bioneer Co., South Korea) and were sent for sequencing (Bioneer Co., South Korea). All sequences from a given sample were combined and used to construct alignments. ClustalX (Version 1.83) multiple sequence alignment analysis was performed to calculate the percentage of sequence similarity between our positive samples and sequences of referral strains and other IBV strains. Phylogenetic trees of sequences were constructed by the neighbor-joining method and the Kimura 2-parameter model by MEGA package, Version 5.1 (15). A bootstrap resampling analysis was performed (1000 replicates) to test the robustness of the major phylogenetic groups.

## RESULTS

**Fig. 1.**
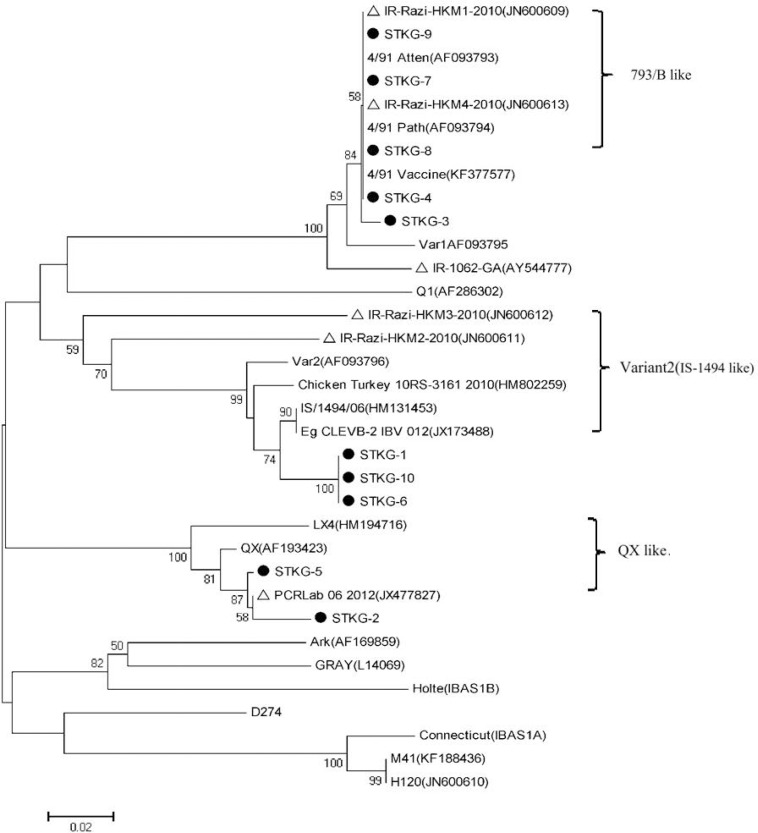
Nucleic acid Phylogenetic relationships of S gene avian infectious bronchitis virus (IBV) detected from backyard chicken in Iran, 2014. The phylogenetic tree was generated using neighboring joining model with MEGA (version 5.1 beta). Numbers below branches indicate bootstrap value from 1000 replicates, bootstrap values. Horizontal distances are proportional to the minimum number of Nucleic acid differences required to join nodes. The vertical lines are for spacing branches and labels. The analysiswas based on complete open reading frames of all gene segments. The scale bar represents the distance unit between sequence pairs. The virus genome characterized in this report is indicated as Black Circle. White triangle is indicated for previous Iranian IBV isolates from commercial poultry farms. The sequences obtained from Gene Bank.

The overall seroprevalence and ELISA titer of IBV antibodies revealed in this study were 54.5% and 4949, respectively. No clinical signs were observed in chickens at the time of blood sampling. The highest mean titer (7597) was observed in Sari, while the lowest (1373) was observed in Amol (Table 1). According to real time PCR results, 31 samples from 75 were IBV genome- positive (Table 2). For spike gene, RT-PCR, using degenerate primers, resulted in amplification of ∼390 bp product. The phylogeny of the IBVs strains isolated in Iran and their relationship to other representative IBV strains were assessed based on sequence analysis of the S gene variable region. Their phylogenetic relationships were compared with IBV sequences available in GenBank and the representatives of the different genotypes. Fig. 1. demonstrates the phylogenetic tree for representatives of each group of IBV isolates and some reference strains of IBV base Spike gene. We detected 3 genotypes: (1)

**Table 2.** The positive rate of positive avian infectious bronchitis viruses (IBV) in Real Time RT-PCR assay in unvaccinated backyard chick-ens in Mazandaran province, Iran, 2014.

**City**	**No. of. Samples**	**Positive (%)**
Behshar	5	60
Sari	10	30
Jouybar	10	30
Neka	10	50
Nour	7	42.8
Babolsar	5	40
Ghaemshahr	8	37.5
Amol	10	50
Babol	10	40
Total & Average	75	41.3

IBV: 793/B (67.7%), (2) Variant 2 (25.8%), and (3) QX (6.5%). Variant 2, QX, and 793/B detected isolates in this study had 100%, 98%, and 99.35% to 100% homology, respectively. Homology percent with some selected references and Iranian IBV isolates based on the spike gene have been summarized in Table 3. Detected QX genotypes had 96.7% to 98.7% similarity with QX stain (AF193423) and 98.3% to 99.67% with Iranian QX strain. Also, Variant 2 genotype had 97.71% homology with IS/1494/06 (HM131453) isolate, and finally 793/B genotype in this study had 99.35% to 100% homology with 4/91 vaccinal and pathogen stains.

**Table 3.** Nucleotide acid sequence homology base spike gene between selected Iranian avian infectious bronchitis viruses (IBV) that detected in backyard chickens and some IBV strains.

	**STKG-1**	**STKG-6**	**STKG-10**	**STKG-2**	**STKG-5**	**STKG-3**	**STKG-4**	**STKG-7**	**STKG-8**	**STKG-9**

	Var2	Var2	Var2	QX	QX	793/B	793/B	793/B	793/B	793/B
4/91 Pathogen (AF09794)	80.28	80.28	80.28	80.28	81.86	99.35	100	100	100	100
4/91Vaccine (KF377577)	80.28	80.28	80.28	80.28	81.86	99.35	100	100	100	100
IS/1494/06 (HM131453)	97.71	97.71	97.71	80.59	83.03	81.14	81.99	81.99	81.99	81.99
Ark (AF169859)	81.12	79.11	81.12	82.01	82.73	78.67	79.11	79.11	79.11	79.11
H120 (JN600610)	81.91	81.91	81.91	80.31	81.46	74.22	75.16	75.16	75.16	75.16
QX (AF193423)	83.59	83.59	83.59	96.71	98.70	81.17	82.01	80.28	80.28	80.28
PCR Lab 06 2012 (JX477827)	82.73	82.73	82.73	98.38	99.67	81.57	82.41	82.41	82.41	82.41
IR-Rzi-HKM2-2010 (JN600611)	82.62	91.27	93.02	80.48	97.67	81.05	81.48	91.27	91.27	95.34
IR-Rzi-HKM1-2010 (JN600609)	80.28	80	80.28	80.28	81.86	99.35	100	100	100	100

## DISCUSSION

Backyard poultry makes approximately 80% of natural flocks in Africa and Asia. Keeping backyard poultry next to other birds in the north of Iran helps the economic level of the families significantly. Based on the Ministry of Agriculture statistics, the number of backyard poultry is 4.51 million. Mazandaran province is the center of Iranian poultry industry, especially in lines, preparents and breeders. There were many reports of high pathogenic avian influenza in backyard and rural bird populations, especially in the north of Iran, so veterinary council inspected the backyard flocks more. Several studies were performed on the serological prevalence of viral agents, such as Newcastle Disease and avian influenza, in backyard poultry (16). The first report of studying infectious bronchitis serology in Iranian backyard flocks was in Isfahan in 2006. The seroprevalence of chicks was 85.3%, while there was no difference in the season of infection (10). In another study on IB serological prevalence of indigenous chicken flocks in the southwest of Iran, 68% prevalence and 1427 titer average were announced using IDEXX kit (17). The mean titer of chickens was around 4949 in this area. The studied chicks showed no clinical signs and vaccination history based on veterinary history and owner claims. The IBV seroprevalence observed in different cities were highly variable (14%–100%), which could have several explanations. It might be expected that chickens located in towns around which commercial farms are situated would have higher prevalence owing to more frequent contact with vaccine strains used in commercial enterprises, but the results with naive backyard chickens indicated that this was not the case. The serology results indicated that IB virus is rotating in the population. Therefore, more attention should be paid to the biosecurity of the domestic village chickens and commercial poultry flocks when the villagers are employed. To date, no study has been performed on the molecular prevalence and genotyping of IBV in backyard chickens in Iran. This study was the first comprehensive research on backyard chickens (Serological & Molecular Study) in Iran. The infection rate was 41.3% based on molecular surveillance. According to geno-typing and phylogenetic results, 793/B was the dominant genotype in this study. Approximately 67.7% of the positive samples belonged to 793/B. Akbariazad et al. first reported 793/B and then it was continued to be reported by different researchers. The results were similar to those of Al-Shekaili et al. study in backyard poultry in Oman (11). Various forms of 793/B vaccines (IB88, 4/91) are used in industrial flocks of Iran. The first isolate studied in 2002 in Iran indicated the severity of the strain (18). 793/B strains are similar to wild and vicinal strains. It may also be possible that the flocks were infected with rotating vaccine strains. To detect this, full - length S gene sequencing was recommended. In addition, it was possible that the virus detected in cecal tonsils upon fixation was the field virus. The interesting point of these studies was the investigation of QX genotype in backyard poultry. QX serotype was first reported in China in 1994. The first reports of QX in Iran industrial poultry farms date back to 2003 (19). This was the first QX tracing in Iran backyard poultry, showing the role of these birds in expanding QX. Finally, we detected Variant 2 genotypes in the backyard populations, which occurred in the Middle East first with reports from different countries of this region, such as Iran, Egypt, and Palestine, with renal tropism (20, 21). The prevalence of Variant 2 was 25.8% in this study and had high homology with Variant 2 viruses that were circulating in commercial flocks of Iran. Cecal tonsil samples were used in this study with acceptable results. The organ could be suggested as an appropriate sample for the molecular study on IBV. It is suggested that sampling of backyard poultry be conducted in a more expanded way in different provinces of Iran to complete the data regarding IB epidemiology. In addition, it would be useful to study molecular surveillance of coronavirus on other species such as ducks and turkeys in this area and other villages of Iran.
